# Nicotine Dependence among College Students Uninterested in Smoking Cessation during the COVID-19 Pandemic: A Cross-Sectional Survey

**DOI:** 10.3390/ijerph20065135

**Published:** 2023-03-14

**Authors:** Makoto Aoike, Yukihiro Mori, Yuka Aoyama, Mamoru Tanaka, Hana Kozai, Yukihiro Shigeno, Hatsumi Kawamura, Masato Tsurudome, Morihiro Ito

**Affiliations:** 1 Graduate School of Life and Health Sciences, Chubu University, 1200 Matsumoto-cho, Kasugai 487-8501, Aichi, Japan; 2 Center for Nursing Practicum Support, Chubu University, 1200 Matsumoto-cho, Kasugai 487-8501, Aichi, Japan; 3 Department of Clinical Engineering, College of Life and Health Sciences, Chubu University, 1200 Matsumoto-cho, Kasugai 487-8501, Aichi, Japan; 4 Department of Food and Nutritional Sciences, College of Bioscience and Biotechnology, Chubu University, 1200 Matsumoto-cho, Kasugai 487-8501, Aichi, Japan; 5 The Fire Department Headquarters in Kasugai-City, Kasugai 486-0856, Aichi, Japan; 6 Department of Biomedical Sciences, College of Life and Health Sciences, Chubu University, 1200 Matsumoto-cho, Kasugai 487-8501, Aichi, Japan

**Keywords:** COVID-19 pandemic, nicotine dependence, KTSND, FTND, smoking students, college students

## Abstract

This study investigated nicotine dependence among Japanese university students who had reached the smoking age (20 years or older) by the time of the coronavirus disease 2019 (COVID-19) pandemic and examined factors that encourage early smoking cessation. Social dependence on nicotine was evaluated using the Kano Total Social Nicotine Dependence Level (KTSND), and physiological dependence was evaluated using the Fagerström Nicotine Dependence Index (FTND). Of the 356 college students who smoked (4.4% of the total), 182 (51.1%) stated that they were not interested in quitting. Furthermore, 124 (68.1%) of those with no interest in quitting smoking were aware that smoking is a high-risk factor for COVID-19, and 58 (31.9%) were unaware. The group not aware of this risk had significantly higher KTSND scores than the group aware of it. The examination of cigarette type that indicated the users of non-conventional cigarette products and dual-user groups scored significantly higher than the cigarette group on FTND items. Overall, the smokers scored above the normal range for social nicotine dependence, suggesting the need to reduce nicotine dependence to encourage college students who continue to smoke to quit smoking.

## 1. Introduction

Smoking increases and exacerbates the risk of contracting various diseases, including malignancies, heart diseases, and cerebrovascular diseases. Smoking cessation is essential to reduce the risk of diseases caused by smoking. A 95% reduction in smoking reduces the risk of cardiovascular disease by only 50% [1]; however, long-term quitters have a reduced risk of malignancy [2], and smoking cessation after a stroke reduces recurrence and death after 5 years [3]. 

The novel coronavirus disease 2019 (COVID-19), caused by severe acute respiratory syndrome coronavirus 2 (SARS-CoV-2), is a respiratory disease that was first reported in Wuhan, China in December 2019 and is responsible for a global pandemic [4]. As of 13 January 2023, the World Health Organization reported approximately 660 million confirmed cases and more than 6.7 million deaths worldwide. Smoking is a risk factor for COVID-19. The use of cigarettes and e-cigarettes damages the respiratory system [5,6], and smokers have been reported to have a higher risk of exacerbating COVID-19 than lifelong non-smokers [7].

New tobacco products, such as e-cigarettes and heated tobacco products, which are replacing conventional cigarettes, are gaining popularity and increasing in users due to misperceptions about their safety. Although e-cigarettes have been developed and marketed as a healthier alternative to conventional cigarettes, there is growing evidence that their aerosols contain numerous poisons, carcinogens, and organic compounds produced by the thermal decomposition of solvents—although in smaller amounts than those derived from conventional cigarettes [8]. Heated tobacco products are also a medium for nicotine delivery and provide an alternative to conventional cigarettes [9]. They are marketed to consumers as less-harmful alternatives to conventional cigarettes [10]. However, aerosols from heated tobacco products contain various harmful components, albeit in smaller quantities than aerosols from combustible materials [11,12]. Similar to the United States [13] and the United Kingdom [14], the use of e-cigarettes is increasing in Japan [15], where approximately 3.1 million people use IQOS-heated tobacco products (Philip Morris International, State of Connecticut, Stamford, CT, USA), 4 million use e-cigarettes, and 2.9 million are dual users [16]. Dual use is associated with higher exposure to toxic substances at levels equal to or greater than those of conventional cigarettes alone [17]. Additionally, dual use may increase nicotine dependence, prolong smoking, and prevent smoking cessation [18]. The dual use of e-cigarettes and conventional cigarettes is significantly associated with the occurrence of respiratory symptoms compared to the use of only one [19]. Despite this, Japanese surveys show that dual users are increasing [20]. The use of newer types, such as e-cigarettes, may aid in attempts to quit smoking [21]. However, there are reports that e-cigarette and heated cigarette use among youths are not significantly associated with attempts to quit smoking [22], and these discrepancies may vary from country to country [22].

Previous studies have reported no significant changes in the smoking status of students during the COVID-19 pandemic [23]. Our previous study also found that approximately 50% of smokers reported no interest in quitting, and two-thirds reported that the number of cigarettes smoked remained unchanged during the COVID-19 pandemic [24]. Moreover, during the COVID-19 pandemic, college students were less interested in quitting smoking and less likely to change their smoking behavior regardless of the smoking device used, even though they were aware of the higher risk of COVID-19 infection and severity [24]. Therefore, it is necessary to examine the factors that contribute to smoking continuation during the COVID-19 pandemic. In addition, the current COVID-19 pandemic needs to be viewed as an opportunity to motivate smokers to implement smoking cessation, and further education on smoking cessation for smokers using all types of smoking devices is a challenge. It has long been noted that college students during the COVID-19 pandemic may have increased nicotine dependence [25]. In addition, an association between anxiety and nicotine dependence among college students during the COVID-19 pandemic has been reported [26]. However, no studies have evaluated the perceived risk of smoking in relation to COVID-19—and smoker nicotine dependence by each smoking device—among college students who have no interest in quitting smoking, even during the COVID-19 pandemic. Therefore, this study aimed to clarify the perception of COVID-19 as a risk factor among college students who were indifferent to smoking cessation and to determine the degree of nicotine dependence using the Kano Test for Social Nicotine Dependence (KTSND), which measures social nicotine dependence, and the Fagerström Test for Nicotine Dependence (FTND), which measures physical nicotine dependence. In Japan, the minimum age for smoking is 20 years, and this is precisely the time when university students reach this age. In order to enhance smoking cessation education, we believe it is important to survey the nicotine dependence level of university students who have just started smoking. We believe that this study is novel in that it examines nicotine dependence from social and physical aspects in college students who were less willing to quit smoking during the COVID-19 pandemic, and also assesses nicotine dependence according to risk perceptions of COVID-19 and smokers’ smoking devices.

## 2. Materials and Methods

### 2.1. Participants

**Figure 1:**
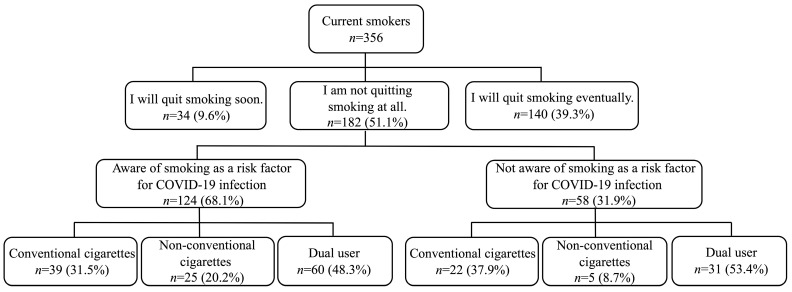
Flowchart of the participants, between March and April 2021, who were undergraduate and graduate students affiliated with a single university in Aichi Prefecture and participated in the questionnaire survey. Conventional cigarettes, cigarettes smokers; non-conventional cigarettes, smokers of heated tobacco products or e-cigarettes; dual users, smokers who use both conventional cigarettes and non-conventional cigarette products.

We conducted a cross-sectional survey with 8547 undergraduate and graduate students affiliated with a single university in the Aichi Prefecture from March to April 2021. The university has many faculties, including humanities, social sciences, natural sciences, health sciences, and engineering. In total, 8117 students (95%) responded to the survey. All respondents met the analysis criteria because there were no missing responses required for the analyses. The flowchart of the participants is presented in Figure 1. Of the 8117 respondents, 356 were current smokers and 182 (51.1%) were not interested in quitting smoking [24]. A total of 182 respondents who indicated that they were not interested in quitting smoking were included in the analyses. The results of the analyses showed that the statistical power in this study was 0.62. The survey was conducted during the third and fourth waves of the COVID-19 pandemic in Japan. During this period, the Japanese government lifted the state of emergency that restricted the movement of its citizens.

### 2.2. Survey Items

#### 2.2.1. Items Related to Attributes and Smoking

The participants were asked about their age, gender, and smoking device. Smokers were categorized as conventional cigarette smokers, users of non-conventional cigarette products, or dual users. In this study, “conventional cigarettes” were defined as cigarettes, and “non-conventional cigarette products” were defined as heated tobacco products or e-cigarettes. Dual users were defined as those using both conventional cigarettes and non-conventional cigarette products. Respondents were asked about their interest in smoking and whether they were aware that smoking is a high-risk factor for COVID-19. The respondents were also asked to select their interest in quitting smoking from one of the following options: “I will quit smoking soon”, “I am not interested in quitting smoking at all”, and “I will quit smoking eventually”.

#### 2.2.2. KTSND

The KTSND is used to quantify social nicotine dependence by assessing the psychological aspects of smoking [27] and is a useful measure for selecting cessation methods and predicting the outcome of smoking cessation treatment [28]. Originally, the KTSND was a scale used by physicians who were active in promoting smoking cessation, and it consists of beliefs about smoking extracted from conversations with smokers as well as words and actions related to beliefs that prevent smoking cessation [27]. Higher scores indicate a greater tendency to justify smoking and deny its harmfulness, and the scale can be used to ascertain perceptions and psychological acceptance of smoking [27]. Table 1 presents the questions and the distribution of scores. The 10 statements in the KTSND questionnaire are as follows: (1) “smoking is a disease in itself”, (2) “smoking is a part of culture”, (3) “smoking is one of life’s pleasures”, (4) “a smoking lifestyle should be respected”, (5) “some people’s lives are enriched by smoking”, (6) “smoking has physical or mental benefits”, (7) “a cigarette is a stress reliever”, (8) “cigarettes enhance the function of smokers’ brains”, (9) “doctors exaggerate the harm of smoking”, and (10) “a place with an ashtray is a place where one can smoke”. The response options are “definitely no”, “probably no”, “probably yes”, and “definitely yes”, with scores of 0, 1, 2, and 3, respectively, for a total score of 30 (reverse scoring was applied for Question 1). A score of 0–9 is within the normal range. In a previous study, the Cronbach’s alpha coefficient for this scale was 0.82, validating its reliability and generalizability [29]. In the present study, the Cronbach’s alpha was 0.89, indicating a good degree of reliability.

#### 2.2.3. FTND

The FTND was adapted from the Fagerström Tolerance Questionnaire [30] and is highly correlated with physiological dependence on cigarettes. Table 2 presents the questions and the distribution of the scores. The questions are (1) “How many cigarettes per day do you smoke?” (2) “How soon after you wake up do you smoke your first cigarette?” (3) “Do you find it difficult to refrain from smoking in places where it is forbidden?” (4) “Which cigarette would you hate most to give up?” (5) “Do you smoke more frequently during the first hours after waking than during the rest of the day?” and (6) “Do you smoke when you are so ill that you are in bed most of the day?” A score of 0–3 indicates low dependence, 4–6 indicates moderate dependence, and 7–10 indicates high dependence [31]. In a previous study, the Cronbach’s alpha coefficient for this scale ranged from 0.56–0.92, validating its reliability and generalizability [32]. The Cronbach’s alpha coefficient in the present study was 0.80, indicating a good degree of reliability.

### 2.3. Statistical Analyses

A *t*-test was used to compare the KTSND and FTND scores after dividing the respondents into two groups: “aware” and “unaware” that smoking is a risk factor for COVID-19 and its severity. A one-way analysis of variance was used to compare KTSND and FTND scores among the three groups according to the smoking device. Bonferroni’s method was used for multiple comparisons. SPSS statistics version 28 (IBM Corp., Armonk, NY, USA) was used for all analyses, and statistical significance was set at less than 5% (*p* < 0.05).

## 3. Results

### 3.1. Basic Attributes

The basic attributes of the participants (*n* = 182) are listed in Table 3. Regarding the type of smoking device used, 61 (33.5%) participants used conventional cigarettes, 30 (16.5%) used non-conventional cigarettes, and 91 (50.0%) dual-used cigarettes. The mean (standard deviation) age was 20.8 (±1.51) years. Of the 182 participants, 124 (68.1%) were aware that smoking was a COVID-19 high-risk factor and 58 (31.9%) were not.

### 3.2. Comparison of KTSND and FTND by Awareness of Smoking as a Risk Factor for COVID-19

**Figure 2:**
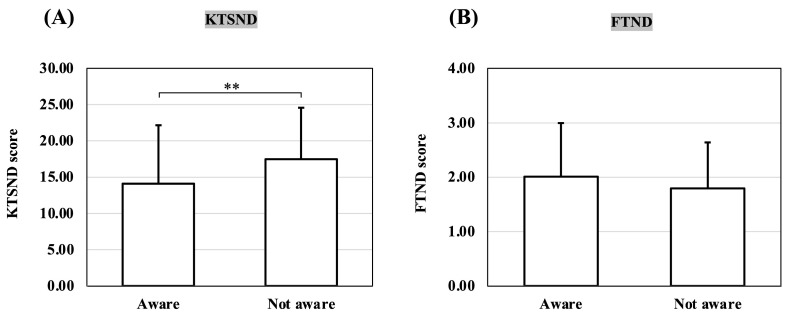
(**A**) Comparison of KTSND scores by awareness of smoking as a risk factor for COVID-19; (**B**) comparison of FTND scores by awareness of smoking as a risk factor for COVID-19. *p*-values are from *t*-tests. **, *p* < 0.01; KTSND, Kano Test for Social Nicotine Dependence; FTND, Fagerström Test for Nicotine Dependence; COVID-19, coronavirus disease 2019.

Figure 2 shows the KTSND and FTND scores according to participants’ awareness of smoking as a risk factor for COVID-19 infection and disease severity. The KTSND scores were significantly higher in the unaware group than in the aware group (*p* = 0.005). However, the mean scores for both groups were above the normal range, suggesting high social dependence on nicotine.

The FTND scores did not differ between the two groups, and the mean scores for both groups were within the low-dependency range for FTND scores.

### 3.3. Comparison of KTSND and FTND Scores by Smoking Device among Those Aware That Smoking Is a Risk Factor for COVID-19

**Figure 3:**
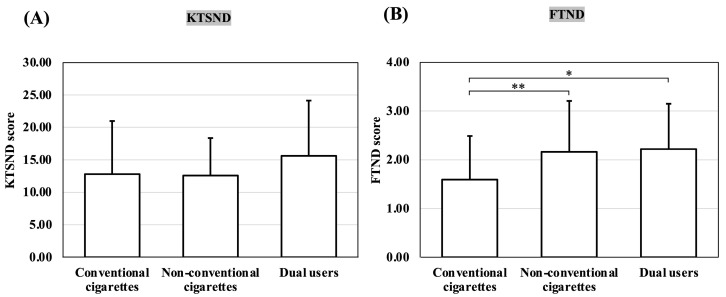
(**A**) Comparison of KTSND scores by smoking device among those who were aware that smoking is a risk factor for COVID-19; (**B**) comparison of FTND scores by smoking device among those who were aware that smoking is a risk factor for COVID-19. All *p*-values are based on multiple comparisons. *, *p* < 0.05; **, *p* < 0.01; KTSND, Kano Test for Social Nicotine Dependence; FTND, Fagerström Test for Nicotine Dependence; COVID-19, coronavirus disease 2019; conventional cigarettes, cigarettes smokers; non-conventional cigarettes, smokers of heated tobacco products or e-cigarettes; dual users, smokers who use both conventional cigarettes and non-conventional cigarette products.

Figure 3 shows the results of the comparisons of KTSND and FTND scores among the 124 participants who were aware that smoking was a risk factor for COVID-19 infection and disease severity based on the smoking device(s) used. The KTSND scores did not differ significantly among smokers based on smoking device use. In addition, the mean KTSND scores were above the normal range for all groups of smoking participants.

The FTND scores for the users of non-conventional cigarette products (*p* = 0.001) and dual users (*p* = 0.012) were significantly higher than those for conventional cigarettes. All *p*-values were based on multiple comparisons. However, the average FTND scores for all smoking groups were within the low-dependency range.

### 3.4. Comparison of KTSND and FTND Scores by Smoking Device among Those Unaware That Smoking Is a Risk Factor for COVID-19

**Figure 4:**
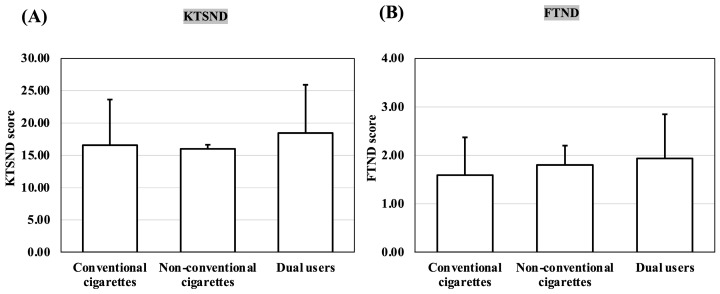
(**A**) Comparison of KTSND scores by smoking device for those unaware that smoking is a risk factor for COVID-19; (**B**) comparison of FTND scores by smoking device for those unaware that smoking is a risk factor for COVID-19. KTSND, Kano Test for Social Nicotine Dependence; FTND, Fagerström Test for Nicotine Dependence, COVID-19, coronavirus disease 2019; conventional cigarettes, cigarettes smokers; non-conventional cigarettes, smokers of heated tobacco products or e-cigarettes; dual users, smokers who use both conventional cigarettes and non-conventional cigarette products.

Figure 4 shows the comparison of the KTSND and FTND scores by smoking device for the 58 participants who were unaware that smoking is a risk factor for COVID-19 infection and disease severity. The total KTSND and FTND scores were not significantly different among the three groups. The mean KTSND score was above the normal range in all groups. In contrast, the mean FTND scores were within the low-dependence range of the FTND scores for all groups.

## 4. Discussion

We conducted a questionnaire survey including university students affiliated with a single Japanese university during the third and fourth waves of the COVID-19 pandemic in Japan. The results of the KTSND demonstrated that the participants who were unaware that smoking is a risk factor for COVID-19 infection and severity were more socially nicotine dependent than those who were aware, indicating that the social nicotine dependence of the former was higher than the latter, although the KTSND score of the former was far above the normal range. However, the result of the FTND revealed that the low physical nicotine dependence of the participants was irrespective of the awareness of the risk. Notably, among the smokers who were aware of the risk, users of non-conventional cigarette products and dual users had higher physical nicotine dependence levels than conventional cigarette smokers. Overall, social nicotine dependence was higher among those who were unaware that smoking was a risk factor for COVID-19 and disease severity.

A previous study reported that smokers who wanted to quit smoking after the start of the COVID-19 pandemic perceived higher risk of infection [33]. Although the present study showed that those who perceived smoking as a risk factor for COVID-19 tended to have lower social nicotine dependence, no association was found with physical nicotine dependence. Overall, we suggest that smoking cessation education for smokers who perceive smoking as a low-risk factor for COVID-19 needs to focus on social nicotine dependence.

An analysis of those who were aware that smoking is a risk factor for COVID-19 indicated the users of non-conventional cigarette products and the dual-user group had higher FTND scores compared to the cigarette group. Since dual use has been reported to be strongly associated with younger age, attempts to quit smoking, and heavy smoking [34], it is possible that, because the college students in this study were younger but old enough to legally smoke, they had less interest in quitting smoking and quitting behaviors. In our previous study, “influence from friends, classmates, and seniors” and “curiosity” were the main reasons for smoking [24]. Although this study did not examine how smokers came to dual use, youths are reported to be more attracted to the taste and appearance of non-conventional cigarettes, such as e-cigarettes, than to the motivation to quit smoking [35]. In this study, physical nicotine dependence was higher in the users of non-conventional cigarette products and dual-user groups, a result that should be noted.

In the present study, the group of smokers with less awareness of risk factors did not differ significantly in social and physical nicotine dependence, regardless of their smoking habits. The FTND, which assesses physical nicotine dependence, was lower among the college students in this study, which is consistent with smoker dependence in previous studies [26].

However, it should be noted that the mean social nicotine dependence score was above the reference value for all smoking groups. Thus, motivational incentives to increase knowledge of the risks of smoking for COVID-19 and approaches to social nicotine dependence are needed for users of all smoking devices. Although the KTSND scores were also above the normal range in previous studies, research on the negative effects of smoking and smoking cessation guidance using outpatient smoking cessation clinic data has reported significant reductions in scores [36], and youths who receive expert advice are more likely to quit smoking [37]. Therefore, it is important to provide appropriate smoking cessation education and guidance to college students who have just started smoking, reduce their nicotine dependence, and create a university environment conducive to treatment. Adjunctive nicotine therapy is a form of smoking cessation treatment that includes the use of gum and patches [38], and it was the first pharmacotherapy proven to be effective in treating tobacco dependence [39]. Nicotine administration has been shown to alleviate nicotine and tobacco withdrawal symptoms and the craving for smoking experienced by smokers for days and weeks after quitting [40]. In addition, varenicline, prescribed in smoking cessation clinics, has been found to increase the likelihood of smoking cessation in adults [41], and abstinence is maintained owing to fewer cravings and withdrawal symptoms [42]. Nicotine dependence on cigarettes is one barrier to successful smoking cessation [43]. Thus, young smokers, such as college students, are important targets in smoking cessation interventions [44] and require education about the harms of nicotine as well as guidance in transitioning to smoking cessation treatments that have been proven effective for smoking cessation. In particular, the findings of high social nicotine dependence among college students who are not interested in quitting smoking and high physical nicotine dependence among users of non-conventional cigarette products and dual users may contribute to developing smoking cessation treatment measures for college students who smoke.

Especially during the COVID-19 pandemic, college students may be more susceptible to nicotine dependence due to anxiety, increasing the necessity and importance of examining the nature of smoking cessation education [26]. During the COVID-19 pandemic, nicotine dependence may be modified due to a variety of factors, including behavioral changes, which may increase smoking dependence [25]. Therefore, along with risk education for smoking during the COVID-19 pandemic, individualized interventions may be necessary to accommodate the diversifying lifestyles of college students. It is also believed that there is a need to ensure that university education provides opportunities to convey the health hazards of smoking and the need to quit smoking.

This study had several limitations. This cross-sectional study was conducted at a single university, and the results may not be representative of the general population because different countries and regions have different attitudes, cultures, lifestyles, and social conditions regarding smoking. The participants in this study were limited to college students with a smoking history of a few months to a few years. Furthermore, this study did not distinguish between heated and e-cigarettes among non-conventional cigarettes; nicotine levels vary depending on the smoking device, which may have affected the results. Depending on the smoking device used, the inclusion of nicotine varied, which may have affected the results. In addition, in recent years, a nicotine dependence rating scale (e-FTCD) has been developed for e-cigarette and dual users [45]. Since the purpose of this study was to compare the nicotine dependence of conventional cigarettes, non-conventional cigarettes, and dual users, the same scale (FTND) was used. The FTND can be adapted to measure nicotine dependence in e-cigarette users [46,47]. However, for smokers who use heated tobacco products—as far as we know—that verification is not sufficient. Furthermore, the KTSND is a scale originally developed based on a survey of the Japanese population [27]. At the time the KTSND was developed, social nicotine dependence was noted as a social psychological tendency—common in smoking-accepting societies, including Japan—to underestimate the harms of smoking and to convince people of its positive effects [28]. Therefore, to our knowledge, with respect to the KTSND, its application to users of non-conventional cigarette products has not been clearly verified; however, the KTSND was applied in this study because it is considered a scale that reflects Japanese culture. In addition, prior studies conducted after the COVID-19 outbreak have suggested that increased mental health problems during the pandemic were also associated with increased tobacco use [48]. However, this study did not collect data on depression or anxiety. Finally, the relatively limited sample size of this study did not allow for high power. Regression analyses considering all covariates could not be employed either; however, it is possible that there are factors other than COVID-19 infection and perceived severity or smoking habits that influence nicotine dependence.

The strength of this study is that it assessed the nicotine dependence of university students using two scales: social and physical. Furthermore, the university included in this study is a comprehensive university with faculties in several academic disciplines and students from all over Japan. The results of this study can be used to implement effective smoking cessation education programs for college students who have continued to smoke. Since the COVID-19 pandemic is expected to continue, we believe that another survey should be conducted to further examine the factors that contribute to continued smoking.

## 5. Conclusions

This study found that social dependence on nicotine was higher among those who were unaware that smoking was a risk factor for COVID-19 and disease severity. In addition, while there was no difference in nicotine dependence among those who were not aware of the risk and the type of smoking device, among those who were aware of the risks, users of non-conventional cigarette products and dual users were more physically dependent on nicotine than those who used conventional cigarettes; this suggests the importance of smoking cessation education in reducing nicotine dependence. In other words, in order to enhance smoking cessation education, it is necessary to investigate nicotine dependence. The results of this study may lead to effective smoking cessation education for students who continue to smoke. No studies have investigated KTSND and FTND scores during a novel coronavirus pandemic, and further research on nicotine dependence is warranted. We believe that those involved in public health and medical care should view the COVID-19 epidemic as an opportunity to promote smoking cessation, provide knowledge about the risk of COVID-19 infection and serious illnesses caused by smoking, and administer individualized interventions according to the diversity of lifestyle behaviors and smoking habits among college students. We have also concluded that universities should ensure that students have an opportunity to learn about the health hazards of smoking and the need to quit smoking.

## Figures and Tables

**Table 1:** KTSND questions.

Questions	Choices and Scores			
1: Smoking is a disease in itself.	DN(3)	PN(2)	PY(1)	DY(0)
2: Smoking is a part of culture.	DN(0)	PN(1)	PY(2)	DY(3)
3: Smoking is one of life’s pleasures.	DN(0)	PN(1)	PY(2)	DY(3)
4: A smoking lifestyle should be respected.	DN(0)	PN(1)	PY(2)	DY(3)
5: Some people’s lives are enriched by smoking.	DN(0)	PN(1)	PY(2)	DY(3)
6: Smoking has physical or mental benefits.	DN(0)	PN(1)	PY(2)	DY(3)
7: A cigarette is a stress reliever.	DN(0)	PN(1)	PY(2)	DY(3)
8: Cigarettes enhance the function of smokers’ brains.	DN(0)	PN(1)	PY(2)	DY(3)
9: Doctors exaggerate the harm of smoking.	DN(0)	PN(1)	PY(2)	DY(3)
10: A place with an ashtray is a place where one can smoke.	DN(0)	PN(1)	PY(2)	DY(3)

DN, definitely no; PN, probably no; PY, probably yes; DY, definitely yes; ( ) = each score. Only question 1 is a reversal item.

**Table 2:** FTND questions.

Questions	Options	Scores
1. How many cigarettes per day do you smoke?	31 or more	3
	21–30	2
	11–20	1
	10 or less	0
2. How soon after you wake up do you smoke your first cigarette?	Within 5 min	3
	6–30 min	2
	31–60 min	1
	After 60 min	0
3. Do you find it difficult to refrain from smoking in places where it is forbidden?	Yes	1
	No	0
4. Which cigarette would you hate most to give up?	The first in the morning	1
	All others	0
5. Do you smoke more frequently during the first hours after waking than during the rest of the day?	Yes	1
	No	0
6. Do you smoke when you are so ill that you are in bed most of the day?	Yes	1
	No	0

**Table 3:** Smoking device use by gender of participants (*n* = 182).

	Conventional Cigarettes (*n* = 61)	Non-Conventional Cigarettes (*n* = 30)	Dual Users (*n* = 91)
Male (*n* = 157)	54 (88.5%)	26 (86.7%)	77 (84.6%)
Female (*n* = 25)	7 (11.5%)	4 (13.3%)	14 (15.4%)

Conventional cigarettes, cigarettes smokers; non-conventional cigarettes, smokers of heated tobacco products or e-cigarettes; dual users, smokers who use both conventional cigarettes and non-conventional cigarette products.

## Data Availability

Not applicable.

## References

[1] Hackshaw A., Morris J.K., Boniface S., Tang J.L., Milenković D. (2018). Low cigarette consumption and risk of coronary heart disease and stroke: Meta-analysis of 141 cohort studies in 55 study reports. BMJ.

[2] Saito E., Inoue M., Tsugane S., Ito H., Matsuo K., Wakai K., Wada K., Nagata C., Tamakoshi A., Sugawara Y. (2017). Smoking cessation and subsequent risk of cancer: A pooled analysis of eight population-based cohort studies in Japan. Cancer Epidemiol..

[3] Epstein K.A., Viscoli C.M., Spence J.D., Young L.H., Inzucchi S.E., Gorman M., Gerstenhaber B., Guarino P.D., Dixit A., Furie K.L. (2017). Smoking cessation and outcome after ischemic stroke or TIA. Neurology.

[4] Li Q., Guan X., Wu P., Wang X., Zhou L., Tong Y., Ren R., Leung K.S.M., Lau E.H.Y., Wong J.Y. (2020). Early transmission dynamics in Wuhan, China, of novel coronavirus-infected pneumonia. N. Engl. J. Med..

[5] Wills T.A., Pagano I., Williams R.J., Tam E.K. (2019). E-cigarette use and respiratory disorder in an adult sample. Drug Alcohol Depend..

[6] McConnell R., Barrington-Trimis J.L., Wang K., Urman R., Hong H., Unger J., Samet J., Leventhal A., Berhane K. (2017). Electronic cigarette use and respiratory symptoms in adolescents. Am. J. Respir. Crit. Care Med..

[7] Patanavanich R., Glantz S.A. (2020). Smoking is associated with COVID-19 progression: A meta-analysis. Nicotine Tob. Res..

[8] Goniewicz M.L., Knysak J., Gawron M., Kosmider L., Sobczak A., Kurek J., Prokopowicz A., Jablonska-Czapla M., Rosik-Dulewska C., Havel C. (2014). Levels of selected carcinogens and toxicants in vapour from electronic cigarettes. Tob. Control.

[9] Jankowski M., Brożek G.M., Lawson J., Skoczyński S., Majek P., Zejda J.E. (2019). New ideas, old problems? Heated tobacco products—A systematic review. Int. J. Occup. Med. Environ. Health.

[10] Lüdicke F., Picavet P., Baker G., Haziza C., Poux V., Lama N., Weitkunat R. (2018). Effects of switching to the menthol tobacco heating system 2.2, smoking abstinence, or continued cigarette smoking on clinically relevant risk markers: A randomized, controlled, open-label, multicenter study in sequential confinement and ambulatory settings (part 2). Nicotine Tob. Res..

[11] Bekki K., Inaba Y., Uchiyama S., Kunugita N. (2017). Comparison of chemicals in mainstream smoke in heat-not-burn tobacco and combustion cigarettes. J. UOEH.

[12] Auer R., Concha-Lozano N., Jacot-Sadowski I., Cornuz J., Berthet A. (2017). Heat-not-burn tobacco cigarettes: Smoke by any other name. JAMA Intern. Med..

[13] Cullen K.A., Ambrose B.K., Gentzke A.S., Apelberg B.J., Jamal A., King B.A. (2018). Notes from the field: Use of electronic cigarettes and any tobacco product among middle and high school students—United States, 2011–2018. MMWR Morb. Mortal. Wkly. Rep..

[14] Conner M., Grogan S., Simms-Ellis R., Scholtens K., Sykes-Muskett B., Cowap L., Lawton R., Armitage C.J., Meads D., Schmitt L. (2019). Patterns and predictors of e-cigarette, cigarette and dual use uptake in UK adolescents: Evidence from a 24-month prospective study. Addiction.

[15] Koyama S., Tabuchi T., Miyashiro I. (2022). E-cigarettes use behaviors in Japan: An online survey. Int. J. Environ. Res. Public Health.

[16] Tabuchi T., Gallus S., Shinozaki T., Nakaya T., Kunugita N., Colwell B. (2018). Heat-not-burn tobacco product use in Japan: Its prevalence, predictors and perceived symptoms from exposure to secondhand heat-not-burn tobacco aerosol. Tob. Control.

[17] Goniewicz M.L., Smith D.M., Edwards K.C., Blount B.C., Caldwell K.L., Feng J., Wang L., Christensen C., Ambrose B., Borek N. (2018). Comparison of nicotine and toxicant exposure in users of electronic cigarettes and combustible cigarettes. JAMA Netw. Open.

[18] Martínez Ú., Martínez-Loredo V., Simmons V.N., Meltzer L.R., Drobes D.J., Brandon K.O., Palmer A.M., Eissenberg T., Bullen C.R., Harrell P.T. (2020). How does smoking and nicotine dependence change after onset of vaping? A retrospective analysis of dual users. Nicotine Tob. Res..

[19] Reddy K.P., Schwamm E., Kalkhoran S., Noubary F., Walensky R.P., Rigotti N.A. (2021). Respiratory symptom incidence among people using electronic cigarettes, combustible tobacco, or both. Am. J. Respir. Crit. Care Med..

[20] Harada S., Sata M., Matsumoto M., Iida M., Takeuchi A., Kato S., Hirata A., Kuwabara K., Shibuki T., Ishibashi Y. (2022). Changes in smoking habits and behaviors following the introduction and spread of heated tobacco products in Japan and its effect on FEV(1) decline: A longitudinal cohort study. J. Epidemiol..

[21] Kinouani S., Pereira E., Tzourio C. (2017). Electronic cigarette use in students and its relation with tobacco-smoking: A cross-sectional analysis of the i-share study. Int. J. Environ. Res. Public Health.

[22] Brown C., Nkemjika S., Yankey B., Okosun I. (2021). Alternative tobacco product use and smoking quit attempts among teenagers in the United States: A cross-sectional study. Cureus.

[23] Gallè F., Veshi A., Sabella E.A., Çitozi M., Da Molin G., Ferracuti S., Liguori G., Orsi G.B., Napoli C. (2021). Awareness and behaviors regarding COVID-19 among Albanian undergraduates. Behav. Sci..

[24] Aoike M., Mori Y., Hotta K., Shigeno Y., Aoyama Y., Tanaka M., Kouzai H., Kawamura H., Tsurudome M., Ito M. (2022). Evaluation of Japanese university students’ perception of smoking, interest in quitting, and smoking behavior: An examination and public health challenges during the COVID-19 pandemic. Drug Discov. Ther..

[25] Celik F.G.N., Demirel G. (2022). Impact of a coronavirus pandemic on smoking behavior in university students: An online survey in Turkiye. Turk. J. Pharm. Sci..

[26] Ayran G., Kose S., Kucukoglu S., Aytekin Ozdemir A. (2022). The effect of anxiety on nicotine dependence among university students during the COVID-19 pandemic. Perspect. Psychiatr. Care.

[27] Yoshii C., Kano M., Isomura T., Kunitomo F., Aizawa M., Harada H., Haradam S., Kawanami Y., Kido M. (2006). Innovative questionnaire examining psychological nicotine dependence, “The Kano Test for Social Nicotine Dependence (KTSND)”. J. UOEH.

[28] Otani T., Yoshii C., Kano M., Kitada M., Inagaki K., Kurioka N., Isomura T., Hara M., Okubo Y., Koyama H. (2009). Validity and reliability of Kano Test for Social Nicotine Dependence. Ann. Epidemiol..

[29] Kitada M., Musashi M., Kano M. (2011). Reliability and validity of Kano Test for Social Nicotine Dependence (KTSND), and development of its revised scale assessing the psychosocial acceptability of smoking among university students. Hokkaido Igaku Zasshi.

[30] Fagerström K.O. (1978). Measuring degree of physical dependence to tobacco smoking with reference to individualization of treatment. Addict. Behav..

[31] Heatherton T.F., Kozlowski L.T., Frecker R.C., Fagerström K.O. (1991). The Fagerström test for nicotine dependence: A revision of the Fagerström Tolerance Questionnaire. Br. J. Addict..

[32] Sharma M.K., Suman L.N., Srivastava K., Suma N., Vishwakarma A. (2021). Psychometric properties of Fagerstrom Test of Nicotine Dependence: A systematic review. Ind. Psychiatry J..

[33] Chertok I.R.A. (2020). Perceived risk of infection and smoking behavior change during COVID-19 in Ohio. Public Health Nurs..

[34] Cho M.S. (2021). Factors associated with cigarette, e-cigarette, and dual use among South Korean adolescents. Healthcare.

[35] Patel D., Davis K.C., Cox S., Bradfield B., King B.A., Shafer P., Caraballo R., Bunnell R. (2016). Reasons for current E-cigarette use among U.S. adults. Prev. Med..

[36] Saito M., Nodate Y., Maruyama K., Tsuchiya M., Watanabe M., Niwa S. (2012). Establishment of a practical training program in smoking cessation for use by pharmacists using cognitive-behavioral therapy and the motivational interview method. Yakugaku Zasshi.

[37] Chandrupatla S.G., Tavares M., Natto Z.S. (2017). Tobacco use and effects of professional advice on smoking cessation among youth in India. Asian Pac. J. Cancer Prev..

[38] Berlin I. (2009). Therapeutic strategies to optimize the efficacy of nicotine replacement therapies. COPD.

[39] Edens E., Massa A., Petrakis I. (2010). Novel pharmacological approaches to drug abuse treatment. Curr. Top. Behav. Neurosci..

[40] West R.J., Jarvis M.J., Russell M.A., Carruthers M.E., Feyerabend C. (1984). Effect of nicotine replacement on the cigarette withdrawal syndrome. Br. J. Addict..

[41] Cahill K., Lindson-Hawley N., Thomas K.H., Fanshawe T.R., Lancaster T. (2016). Nicotine receptor partial agonists for smoking cessation. Cochrane Database Syst. Rev..

[42] Coe J.W., Brooks P.R., Vetelino M.G., Wirtz M.C., Arnold E.P., Huang J., Sands S.B., Davis T.I., Lebel L.A., Fox C.B. (2005). Varenicline: An alpha4beta2 nicotinic receptor partial agonist for smoking cessation. J. Med. Chem..

[43] Li H., Zhou Y., Li S., Wang Q., Pan L., Yang X., Zhang N., Jiang F., Han M., Jia C. (2015). The relationship between nicotine dependence and age among current smokers. Iran J. Public Health.

[44] Zhan W., Dierker L.C., Rose J.S., Selya A., Mermelstein R.J. (2012). The natural course of nicotine dependence symptoms among adolescent smokers. Nicotine Tob. Res..

[45] Piper M.E., Baker T.B., Benowitz N.L., Smith S.S., Jorenby D.E. (2020). E-cigarette dependence measures in dual users: Reliability and relations with dependence criteria and e-cigarette cessation. Nicotine Tob. Res..

[46] Etter J.F., Eissenberg T. (2015). Dependence levels in users of electronic cigarettes, nicotine gums and tobacco cigarettes. Drug Alcohol Depend..

[47] Johnson J.M., Muilenburg J.L., Rathbun S.L., Yu X., Naeher L.P., Wang J.S. (2018). Elevated nicotine dependence scores among electronic cigarette users at an electronic cigarette convention. J. Commun. Health.

[48] Tzu-Hsuan Chen D. (2020). The psychosocial impact of the COVID-19 pandemic on changes in smoking behavior: Evidence from a nationwide survey in the UK. Tob. Prev. Cessat..

